# Tolerogenic dendritic cells in radiation-induced lung injury

**DOI:** 10.3389/fimmu.2023.1323676

**Published:** 2024-01-08

**Authors:** Benbo Liu, Yilong Wang, Gencheng Han, Maoxiang Zhu

**Affiliations:** Beijing Key Laboratory for Radiobiology, Beijing Institute of Radiation Medicine, Beijing, China

**Keywords:** radiation-induced lung injury(RILI), tolerogenic dendritic cells(tolDCs), regulatory T cells(Tregs), regulatory B cells(Bregs), inflammation

## Abstract

Radiation-induced lung injury is a common complication associated with radiotherapy. It is characterized by early-stage radiation pneumonia and subsequent radiation pulmonary fibrosis. However, there is currently a lack of effective therapeutic strategies for radiation-induced lung injury. Recent studies have shown that tolerogenic dendritic cells interact with regulatory T cells and/or regulatory B cells to stimulate the production of immunosuppressive molecules, control inflammation, and prevent overimmunity. This highlights a potential new therapeutic activity of tolerogenic dendritic cells in managing radiation-induced lung injury. In this review, we aim to provide a comprehensive overview of tolerogenic dendritic cells in the context of radiation-induced lung injury, which will be valuable for researchers in this field.

## 1 Introduction

Radiation-induced lung injury (RILI) refers to any damage to the lungs caused by exposure to ionizing radiation (1, 2). The recent Fukushima nuclear sewage incident has raised significant concerns regarding its impact on human health (3, 4). Since the lung is a radiation-sensitive organ, nuclear pollution can potentially lead to radioactive damage in the lungs (5, 6). Clinical manifestations of RILI are typically divided into two stages: pneumonia in the early stages and pulmonary fibrosis in the late stages (7–9). However, the pathological process of RILI is complex, and the molecular mechanism underlying its development remains poorly understood. Currently, there are no specific therapeutic drugs available for this condition.

Recently, numerous studies have demonstrated the potential of dendritic cell (DC)-based therapy as a promising treatment for diseases associated with lung injury (10–13). DCs, which are strategically located between the airway epithelial cells and the matrix, are known for their strong antigen-presenting abilities and immune monitoring of the lungs (14, 15). DCs are a heterogeneous group of myeloid-derived cells that can be found in almost all tissues (16, 17). When there is an invasion of pathogens or tissue inflammation, DCs quickly migrate to the damaged tissue site, process antigens, and present them to T lymphocytes, thereby bridging the innate immune response and adaptive immunity (18). Indeed, DCs have diverse functions and plasticities within the immune system. They have been extensively studied in various fields such as autoimmune diseases, inflammation, cancer, and fibrosis in different organs including the lungs, liver, kidneys, and heart (19–21).

Recent studies have also highlighted the role of tolerogenic DCs (tolDCs) in negatively regulating the immune response and maintaining immune tolerance. They promote the elimination of autoreactive immature T-cells in the thymus and induce immune tolerance in the periphery by interacting with T-cells, leading to T-cell deletion, T-cell incompetence, and differentiation of regulatory T-cells (Treg) subgroups (22–24). Based on the available evidence, there has been an increasing interest in exploring the therapeutic potential of tolDCs for the management of radiation-related diseases. Here, we review the latest developments in the potential application and treatment mechanisms of tolDCs in RILI. Specifically, we focused on the immunosuppressive effects of tolDCs, which may represent an important therapeutic advantage in the treatment of RILI and pave the way for clinical trials.

## 2 Pathogenesis of RILI

**Figure 1:**
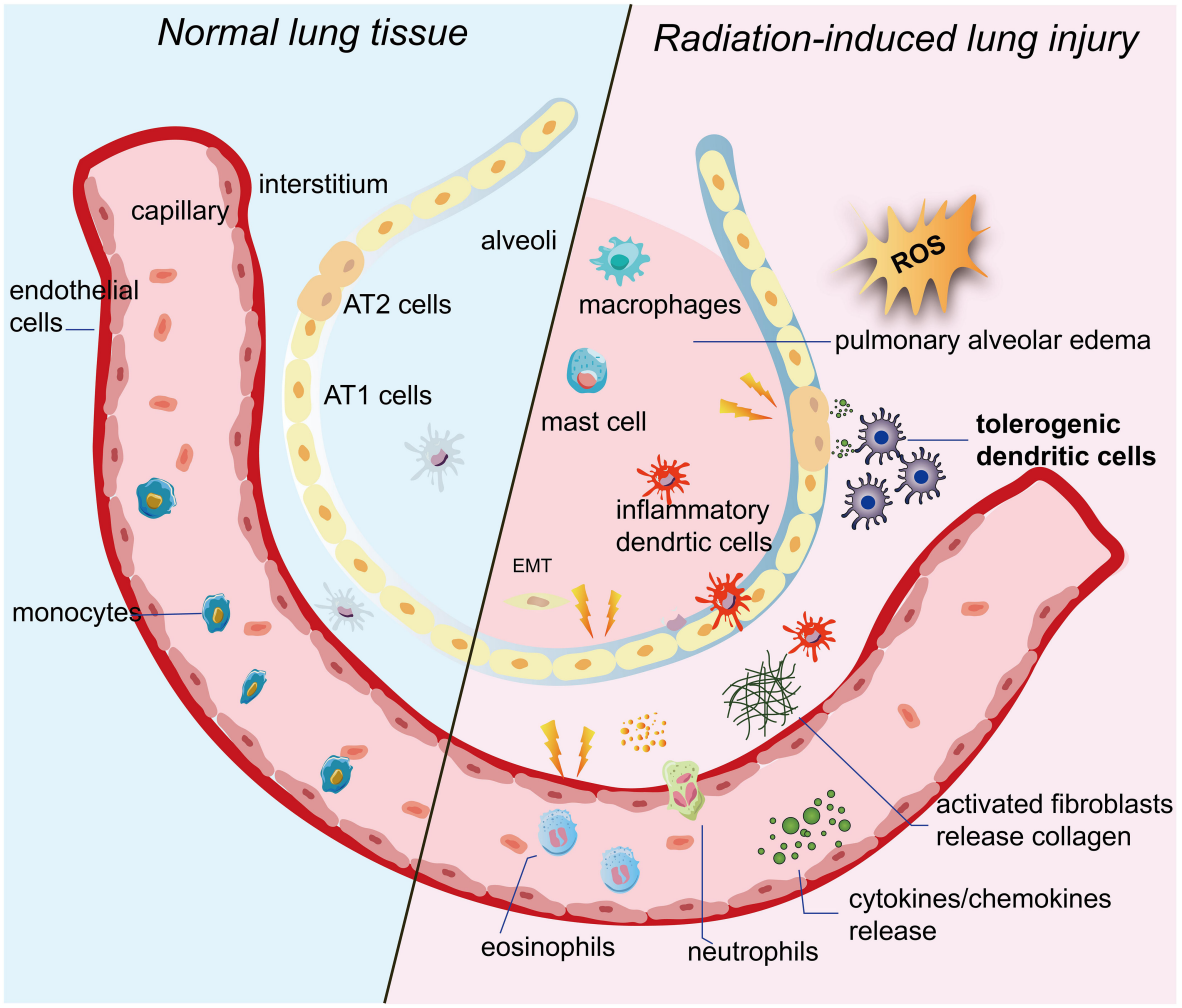
Pulmonary tissue undergoes changes following irradiation. When ionizing radiation is applied to lung tissue, it ionizes water molecules and generates a significant amount of ROS. These persistent ROS can exacerbate damage to target cells such as alveolar epithelial cells and vascular endothelial cells. The damaged target cells then release inflammatory cytokines and chemokines. Additionally, imDCs located between the pulmonary epithelium and interstitium promptly migrate to the site of injury. They not only enhance T-cell activity, but also activate and mobilize other immune cells. As a result, the immune system becomes abnormally activated. Inflammatory cells and fibrosis-associated cells are activated and infiltrate the interstitium. Eventually, the epithelium undergoes the EMT process, and fibroblasts differentiate into myofibroblasts, leading to excessive collagen deposition. Ultimately, this excessive collagen deposition contributes to the development of pulmonary fibrosis. IR, ionizing radiation;ROS, reactive oxygen species; imDCs, immature dendritic cells; AT1 cells, alveolar type 1 cells; AT2 cells, alveolar type 2 cells; EMT, epithelial-mesenchymal transition.

RILI is a complex pathological process that involves multiple cells. Following radiation exposure, acute exudation occurs in the lungs, which is characterized by inflammatory cell infiltration, varying degrees of transparency in small arteries, fibrous thickening, alveolar edema filled with exudate, and collagen fibrous hyperplasia. It is important to note that this process is irreversible (25, 26). However, the precise molecular mechanism of RILI remains unclear. So far, the existing research, both domestic and foreign, has mainly focused on a few key areas. These areas include the generation of reactive oxygen species (ROS), direct damage to target cells, and activation of the immune system, as shown in **Figure 1**.

When ionizing radiation is applied to lung tissue, it ionizes water molecules and generates a significant amount of ROS (27–29). These ROS can damage the DNA, proteins, and lipid membranes of target cells. If the damage is not promptly repaired, it can lead to oxidative stress damage. Research has shown that oxidative stress damage can still be detected weeks or even months after the completion of radiotherapy (30). Moreover, these persistent ROS can worsen the damage to target cells and perpetuate lung lesions (31–33).

According to extensive research, vascular endothelial cells and alveolar epithelial cells have been identified as the main target cells for RILI (34, 35). In the early stages after radiation exposure, vascular endothelial cells exhibit increased vascular permeability and inflammatory exudation (33). As the duration of irradiation and the dose of radiation increase, vascular endothelial cells undergo rupture and detachment, leading to platelet attachment and resulting in capillary embolism and fibrosis (36–38). Type I alveolar epithelial cells, which lack the ability to proliferate, undergo direct necrosis or apoptosis following irradiation. Damage to type II alveolar epithelial cells can trigger excessive proliferation of fibroblasts, leading to fibrosis (39–41). Moreover, the abnormal proliferation of type II alveolar epithelial cells reduces the secretion of alveolar surface-active substances, resulting in decreased alveolar surface tension and causing pulmonary tissue edema and atelectasis (42, 43).

Of note, damaged alveolar epithelial cells and vascular endothelial cells also secrete various cytokines, including tumor necrosis factors (TNF-α) involved in local injury and inflammatory response, transforming growth factor β-1 (TGF-β1) that promotes tissue repair and organ fibrosis, platelet-derived growth factor (PDGF), interleukins (IL-1β, IL-6, IL-8, IL-10), and monocyte chemotactic peptides (32, 44, 45). Among them, some studies have shown that TGF-β1-based CRISPR/Cas9 gene therapy improves lung tissue pathological damage, reduces the secretion and expression of inflammatory factors, and ultimately inhibits the progression of radiation fibrosis (46).

More importantly, the immune system, which serves as the body’s primary defense against external damage, has been extensively studied and found to play a crucial role in the onset and progression of RILI (47). Among the various components of the immune system, DCs are the most potential professional antigen presenting cells and play a dominate role in immune system as commanders during RILI. In the early stages of RILI, immature DCs(imDCs) situated between the pulmonary epithelium and interstitium promptly detect endogenous damage-associated molecular patterns (DAMPs) released from damaged or dying cells through their surface pattern recognition receptors (PRRs) (48–53). They swiftly migrate to the site of injury and initiate an initial immune response by efficiently capturing, processing, and presenting antigens to T-cells in nearby lymph nodes (54). T-cells play a crucial role in the immune system as communication experts. They activate B-cells, which release a large number of antibodies and contribute to the ultimate defense (55–57). Furthermore, DCs secrete cytokines and growth factors to enhance and regulate various immune responses, including those of macrophages, mast cells, NK cells, and cytokine-induced killer (CIK) cells (58–60). Simultaneously, these activated immune cells release high levels of pro-inflammatory cytokines, including IL-1β and TNF-α, as well as chemokines like chemokine C-C-motif ligand 1 (CCL1). These cytokines further stimulate fibroblasts to differentiate into myofibroblasts, leading to excessive collagen deposition (61–63). Ultimately, this excessive collagen deposition contributes to the development of radiation-induced pulmonary fibrosis (64–66).

However, several subsets of DCs have been discovered with the development of DC studies on lung tissue. It is crucial to note that these different subgroups of pulmonary DCs exhibit a division of labor. Therefore, it is necessary to gain a deeper understanding of the biological and functional characteristics of pulmonary DCs to identify a specific subset of DCs that plays a pivotal regulatory role in RILI.

## 3 The phenotype of heterogeneous DC subsets of the lung

DCs are the primary antigen-presenting cells in the lung tissue and are crucial for the immune response to RILI (67–69). Within the lung tissue, DCs are categorized into three main types: conventional DCs, plasmacytoid DCs, and monocyte-derived DCs, as shown in **Table 1**.

**Table 1:** Subsets and functions of pulmonary DC populations.

Subsets	General functions in lung tissue	Transcription factor	Key markers (human)	Ref.
cDC1	Effectively stimulates CD8^+^ T-cells in response;facilitates Th1-assisted T-cells and natural killer responses	BATF3, ZEB2, IRF8, PU.1, FIT3L, ZBTB46, ID2	CD8a, CLEC9A, CD103, CD11c, CD141, XCR1	(70–73)
cDC2	Regulates Th2 and Th17 auxiliary T-cell responses	IRF4, NOTCH2, KLF4, ZEB2, PU.1, FIT3L, ZBTB46, ID2	CD1c, CD207, CD11b, NOTCH2, SIRPα	(74–76)
cDC2A	Exerts anti-inflammatory potential	T-bet, Runx3, SREBF2	CD5	(77, 78)
cDC2B	Exerts pro-inflammatory potential	RORƴt, CEPBA	CD14,CD163	(78–80)
AS DC	stimulates T-cells response	ZEB2, IRF4, IRF8, KLF4, PU.1, FIT3L	AXL,Siglec6 (CD327)	(81, 82)
pDC	Rapidly produce cytokines such as type I and type III interferons; activates CD8^+^ T-cells	IRF4, IRF8, ZEB2, PU.1, FIT3L, TCF4	CLEC4C, LILRB4, NRP1, CCR7, B220 (in mice), SiglecH (in mice)	(83–98)
moDC	Promotes CD4^+^ T-cells to generate a Th17 immune response	MAFB, KLF4	CD14, CD206, CD209, SIRPα, CD11b, CD1a	(99–106)

cDC1, conventional type 1 dendritic cell; BATF3, basic leucine zipper transcriptional factor ATF-like 3; ZEB2, zinc finger E-box binding homeobox 2; IRF8, interferon regulatory factor 8; PU.1, Spi-1 proto-oncogene; FIT3L, Fms-related tyrosine kinase 3 ligand; ZBTB46, zinc finger and BTB domain containing 46; ID2, inhibitor of DNA binding 2; CLEC9A, C-type lectin domain family 9 member A; XCR1,chemokine (C motif) receptor 1; NOTCH2, Notch homolog 2; KLF4, kruppel like factor 4; SIRPα, signal regulatory protein alpha; T-bet, T-box transcription factor, TBX21; Runx3, runt-related transcription factor 3; SREBF2, sterol regulatory element binding transcription factor 2; RORƴt, retinoic acid-related orphan receptor gamma t; CEBPA, CCAAT/enhancer binding protein (C/EBP), alpha; AXL, receptor tyrosine kinase; CLEC4C, C-type lectin domain family 4 member C; LILRB4,Leukocyte immunoglobulin-like receptor subfamily B member 4; NRP1-1, Neuropilin-1; CCR7,  C-C chemokine receptor type 7; TCF4, transcription factor 4; MAFB, transcription factor MafB; pDC, plasmacytoid DC.

Notably, it has been reported that many conventional DCs(cDCs) are located in the mucous membranes of the airway ducts (107–109). These cells extend pseudopods between epithelial cells to capture antigens present within the airway cavity (102, 110, 111). Based on their functional differences, cDCs are further categorized into two subsets: cDC1 and cDC2. Among them, mice and humans cDC1 have been observed to exhibit a high degree of cross-presentation ability, effectively stimulating CD8^+^ T-cells in response to extracellular antigens like those found in bacteria and viruses (70, 71). These cells are known to secrete IL-12, type I, and III interferons (IFNs), and are believed to facilitate Th1-assisted T-cells and natural killer responses (72, 73). Some well-known markers for cDC1 include CD8a, CLEC9A,CD103, CD11c, CD141, and XCR1. Additionally, cDC2, which is the primary subset of DC found in the blood, tissues, and lymphatic organs, has been demonstrated to stimulate Th2 and Th17 auxiliary T-cell responses (74, 75). Moreover, cDC2 has been found to have several regulatory effects, such as the induction of Tregs in lung tissue and the maintenance of tolerance in the same tissue (76). Common markers used to identify cDC2 include CD1c, CD207, CD11b, NOTCH2, and SIRPα. However, recent evidence has accumulated, indicating that cDC2 is not a homogeneous population, but rather consists of two distinct subsets: cDC2A and cDC2B (77). These subsets are differentiated based on the expression levels of two key transcription regulators, T-bet and ROR-ƴt (77). The cDC2A subset demonstrates an anti-inflammatory function (78), while the cDC2B subset exhibits pro-inflammatory properties (78–80). Consequently, the cDC2 subsets have significant implications in maintaining lung tissue homeostasis and regulating immune responses. Of note, AXL^+^Siglec6^+^ DCs (AS DC), the precursor to mature cDC2, can potentially transition to this subset through the influence of two key regulators, AXL and Siglec6 (CD327) (81). It has been reported that this subset strongly stimulates T-cell response in lung tissue (82).

Plasmacytoid DCs (pDCs) undergo direct maturation from common DC progenitors (CDPs) in the bone marrow and subsequently migrate to the blood and surrounding lymphoid tissue (83). These round plasmacytoid cells are present in lower numbers compared to regular cells. pDCs are known for their ability to rapidly produce cytokines such as type I and type III IFNs (84–88), which makes them a crucial subset for managing RILI. pDCs also express major histocompatibility complex II (MHC II) and may function as antigen-presenting cells (89–92). There are different subsets of pDCs, including one population characterized by high expression of CD2, which has been observed to specifically induce CD4^+^ T-cell proliferation (93–95). Additionally, upon stimulation, pDCs have been reported to activate CD8^+^ T-cells (96–98). Currently, the primary markers used to identify pDCs include CLEC4C, LILRB4, NRP1, CCR7, B220 (in mice), and SiglecH (in mice).

Monocyte-derived DCs (moDCs) are a DC subset that has been gaining attention due to their strong impact on adaptive immune function and their rapid accumulation in response to an inflammatory stimulus (99–101). Initial studies have primarily focused on the infection aspect of moDCs. However, their significance in RILI is now being increasingly acknowledged. Indeed, moDCs have been observed in human lung mucosal tissue as well as in inflammatory settings (102). In such settings, they are often referred to as ‘pneumonic DCs’ and are generated from monocytes that are recruited from the blood to the lung tissue during inflammation. Although this particular subpopulation exhibits dendrite morphology, it also possesses the genetic signature of moDCs *in vitro*. Hence, it is generally accepted that moDCs are produced as part of the inflammatory response, and this subgroup plays a role in promoting CD4^+^ T-cells to generate a Th17 immune response (105, 106). Currently, reported moDC markers include CD14, CD206, CD209, SIRPα, CD11b, and CD1a.

Taken together, DCs are heterogeneous populations. Therefore, future research should focus on linking the phenotypic features of different DC subsets to the known functions of their biology, especially when considering clinical applications based on DCs.

## 4 Balancing immunity and tolerance by pulmonary DCs in RILI

**Figure 2:**
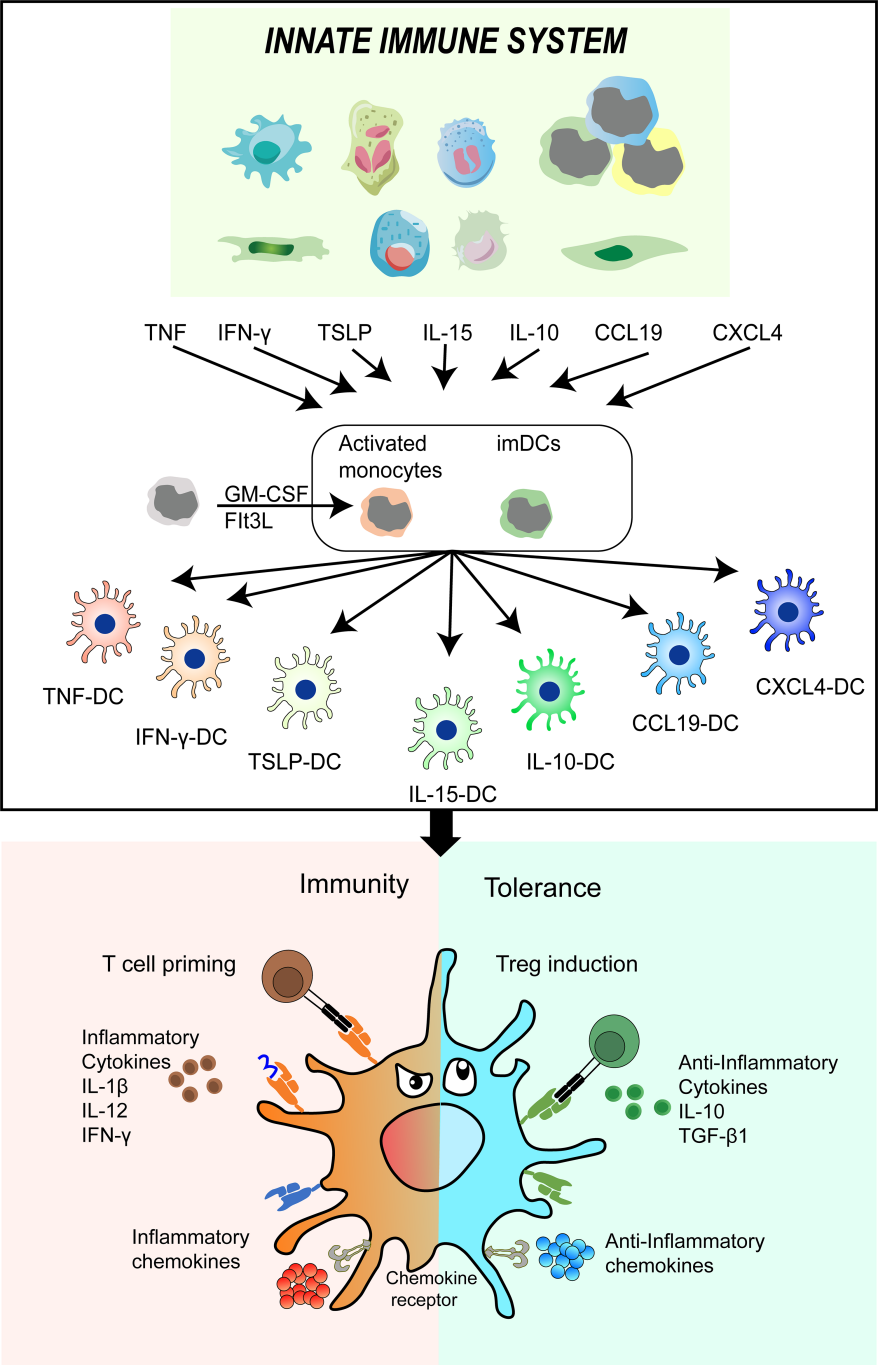
DC phenotypes and functions undergo alterations in response to the microenvironment of lung tissue induced by ionizing radiation. The intrinsic immune cells are stimulated by ionizing radiation to release various soluble factors including TNF-α, IFN-ƴ, TSLP, IL-15, IL-10, CCL19, and CXCL4. These factors subsequently promote the differentiation of imDCs and their precursors into infDCs and tolDCs. This cascade of events further impacts other immune cells, ultimately disrupting the delicate balance between immunity and tolerance. DC, dendritic cell; IR, ionizing radiation; TNF-α, tumor necrosis factors; IFN-ƴ, interferons gama; TSLP, thymic stromal lymphopoietin(TSLP); IL-10, interleukin 10; CCL19, chemokine C-C-motif ligand 19; CXCL4, chemokine CXC-motif ligand 4; imDCs, immature DCs; infDCs, inflammatory DCs; tolDCs, tolerogenic DCs.

DCs play a crucial role in maintaining a balance between immunity and tolerance (112–114). They act as messengers in the occurrence and development of respiratory diseases (115–117). Additionally, they serve as sensors and tolerant gatekeepers for airway mucosa pathogens (118–120). This multifunctional cell type combines innate signaling mechanisms such as pattern recognition and early inflammatory mediators with adaptive immune responses, including T-cell priming and Treg induction (121, 122). Ionizing radiation disrupts the balance between effector immunity and regulatory immunity in lung tissue, leading to alterations in the behavior of DCs in response to these changes (**Figure 2**).

Ionizing radiation induces a cascade of cellular and molecular changes in lung tissue, leading to the release of numerous cytokines, chemokines, and growth factors (123–125). These substances attract immune cells, resulting in the formation of a microenvironment within the lung tissue. Within this microenvironment, immature DCs (imDCs) and their precursors can be activated by various factors, including those from intrinsic immune cells, to differentiate into mature cells with distinct phenotypes and functions (50, 126). Therefore, TNF-α, IFN-γ, thymic stromal lymphopoietin(TSLP), IL-15, IL-10, chemokine C-C-motif ligand 19 (CCL19) and chemokine CXC-motif ligand 4(CXCL4) differentiated DCs into TNF-DCs, IFN-DCs, TSLP-DCs, IL-15-DCs, IL-10-DCs, CCL19-DCs or CXCL4-DCs, respectively. These distinct phenotypes and functions of DCs play a crucial role in shaping different types of T-cell immunity. For example, TSLP-DCs develop T-cells into inflammatory type 2 cells, secreting large amounts of TNF and type 2 cytokines (127). IL-10-DCs promote the development of IL-10-secreted Treg (128). IFN-γ-DCs promote effective T-cell response by upregulating IL-12 secretion (129). CXCL4-DCs enhance the proliferation of autologous CD4^+^ T-cells and CD8^+^ T-cells and the production of IFN-γ and IL-4 (130, 131).

In light of the available evidence, we suggest that imDCs and their precursors possess remarkable functional plasticity, enabling the intrinsic immune system to modulate the specific immune system. Additionally, this process gives rise to two types of DCs: inflammatory DCs (infDCs) that initiate positive immune cell responses (132, 133), and tolDCs, which elicit negative regulatory immune responses and contribute to the maintenance of immune tolerance (134). As a result, the outcome of various inflammatory diseases is determined by the conflict between the exaggerated immune response activated by infDCs and the immune response negatively regulated by tolDCs. Collectively, conducting comprehensive research on the potential application mechanisms of tolDCs could offer a promising and innovative approach to treating RILI.

## 5 Potential mechanisms of tolDC-based therapy for RILI

**Figure 3:**
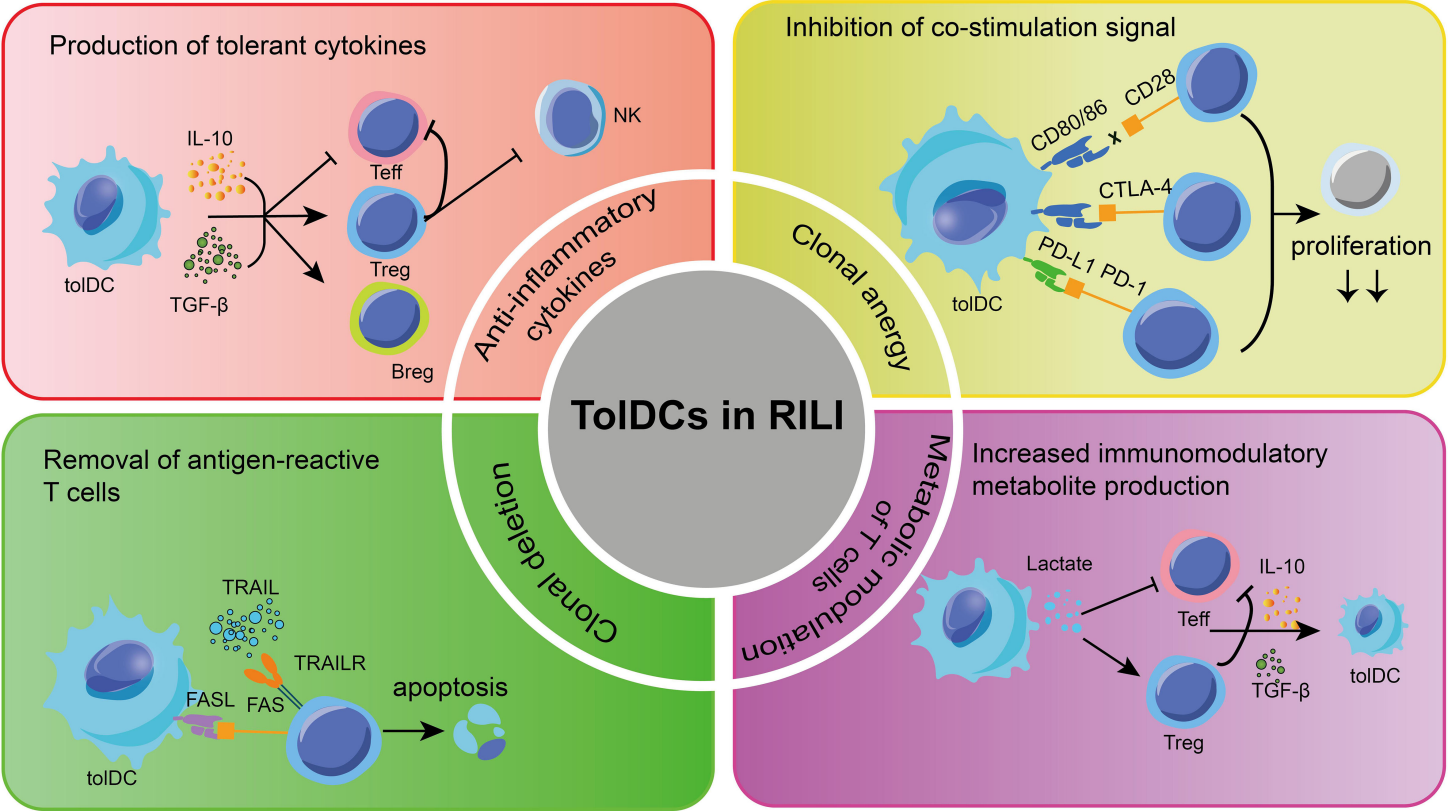
Some mechanisms about tolDCs for RILI in current research. The main methods to apply tolDCs in RILI are shown in the picture. TolDCs can regulate multiple signaling molecules, which protect pulmonary cells. TolDCs inhibit leukocyte infiltration and regulate leukocyte function. TRAIL, TNF-related apoptosis-inducing ligand; FasL, fas ligand; TGF-β, transforming growth factor beta; Treg, regulatory T-cells; Breg, regulatory B cell; Teff, effector cells;PD-L1, programmed cell death 1 ligand 1; PD-1, programmed cell death protein 1;CTLA4, cytotoxic T-lymphocyte-associated protein 4.

Accumulating clinical studies have demonstrated that breast cancer patients who receive unilateral chest wall radiotherapy exhibit a notable presence of activated lymphocytes in bilateral alveolar lavage (135). This finding suggests that the observed inflammatory change in RILI is not solely caused by tissue injury, but rather involves an exaggerated immune response of T and B-cells. Specifically, infDCs activate multiple immune cell inflammatory response processes in various diseases. Initially, the focus of understanding DCs in RILI was on this group. However, recent evidence indicates that tolDCs also play a crucial role in regulating inflammation and preventing excessive immune response-induced damage to lung tissue. Therefore, there is potential for utilizing tolDCs to address immune disorders in RILI through the following aspects (**Figure 3**):

TolDCs play a significant role in suppressing immune responses through various mechanisms, including the production of cytokines and cell-cell contact. Several studies have demonstrated that certain anti-inflammatory cytokines, including TGF-β and IL-27, secreted by various tolDCs, play a role in promoting the production of Tregs and stimulating the secretion of IL-10 (136). Besides, tolDC also promotes the Bregs to produce IL-10, TGF-β, and to some extent, IL-35, and further inhibit antigen-specific CD8^+^ T-cells in inflammation and autoimmune diseases (137–139). On the other hand, growing evidence has demonstrated that tolDCs exhibit limited capacity for cross-expression and low co-stimulation molecular phenotypes (140–142). In the absence of these co-stimulation signals, T-cells are unable to produce IL-2 and undergo further proliferation when they interact with tolDCs through the recognition of antigens presented by MHC via T-cell receptors (TCR) (143). As a result, the overreaction of T-cells in RILI is eventually blocked.

Of note, tolDCs express programmed cell death 1 ligand 1(PD-L1) and PD-L2, which bind with programmed cell death protein 1 (PD-1) in T-cells (144, 145). This interaction leads to the recruitment of SH2-containing inositol phosphatase 1(SHP-1) and SHP-2, instead of activating the TCR and CD28 signaling pathway. As a result, tolDCs induce clonal incompetence and promote the differentiation of Tregs, ultimately leading to tolerance in RILI. Furthermore, the study revealed that the interaction between TNF-related apoptosis-inducing ligand (TRAIL) in human DCs and T-cell death receptors can induce T-cell apoptosis by activating caspase. Likewise, the fas ligand (FasL) present on the surface of tolDCs binds to the upregulated Fas during T-cell activation, thereby promoting T-cell apoptosis (146).

In general, these findings demonstrate that tolDCs are capable of interacting with Tregs and Bregs, thus forming a feedback loop of tolerance. This means that once a regulating group initiates a tolerance signal, it can be sustained and enhanced through the aggregation of other cell groups. Therefore, tolDCs can be considered as a potential treatment option for radioactive lung injury, particularly during the initial stages of pneumonia. However, limited information is available regarding the origin of tolDCs.

## 6 *In vivo* and *in vitro* generated tolDCs

In 1998, Steinman made a discovery that highlighted the dual role of DCs. While primarily involved in T-cell immune responses, DCs also have the paradoxical effect of inducing tolerance to autoantigens (147, 148). Subsequent research has revealed that a specific subset of DCs, known as tolDCs, possess functions that can inhibit T-cell profiles or induce Treg (149). According to domestic and foreign reports, the acquisition of tolDCs primarily involves the following aspects:

First, the stromal environment, particularly in the bone marrow, spleen, lung, kidney, and liver, contributes to the induction of tolDCs. The study revealed that co-culturing DCs with spleen stromal cells resulted in a negative immune function (150). Furthermore, co-culturing mesenchymal stem cells (MSCs) with imDCs or mature DCs (mDCs) resulted in the generation of a distinct subset of tolDCs (150). These tolDCs exhibited reduced expression of costimulatory molecules, decreased levels of IL-12, and increased levels of TGF-β and IL-10. Importantly, these changes were not reversed when the tolDCs were stimulated with lipopolysaccharides (LPS), indicating sustained immune toleration (151, 152).

Second, some drugs or chemical agents have been found to induce tolDCs. These drugs including immunosuppressants like sirolimus, tacrolimus, cyclosporin, and motidimethylphenol, have been shown to affect the expression of molecules such as CD40, CD80, and CD86 on the surface of DCs, with sirolimus having the most significant impact (144, 153, 154). In addition, numerous studies have shown that the immunomodulatory effect of vitamin D3 is achieved by binding to the vitamin D receptor expressed by DCs, whose immunosuppressive effect manifested as down-regulation of CD80, CD86, CD40, lower IL-12, and higher IL-10, thus inhibiting differentiation and maturation of DCs (155–158).

Third, genetic engineering technology was employed to modify the genes of DCs, preventing their transformation into mDCs and inducing them to secrete inhibitory cytokines, thereby functioning as tolDCs. The previous study has found that *in vitro*, adenovirus vectors are used to modify imDCs, which expressed cytotoxic T lymphocyte-associated antigen-4 (CTLA-4) and low levels of CD86, eventually differentiated into tolDCs (140, 159–163).

Fourth, it has been observed that anti-inflammatory cytokines or chemokines have the ability to stimulate imDCs or DC precursor cells to differentiate into tolDCs. The study has shown that the induction of anti-inflammatory cytokines, such as IL-37, TGF-β, prostaglandin E2 (PGE2), and TNF-α, can stimulate the enzyme activity of indoleamine 2,3-dioxygenase (IDO) in DCs, resulting in the generation of tolDCs (164–168). In addition, recent studies have shown that anti-inflammatory chemokines can transform into tolDCs by binding to their receptors. Azzaoui et al. (169) discovered that the chemokine CCL18, through an IL-10-mediated mechanism, is dependent on the production of IDO to differentiate DCs into tolerogenic cells capable of activating Tregs.

Taken together, with the recognition of tolDCs, there is an increasing focus on finding a quick and efficient acquisition method. This method will play a crucial role as a therapeutic tool in the future. The successful application of tolDCs in the clinical treatment of RILI will depend on the rational use and selection of appropriate access.

## 7 Conclusion

RILI is a serious and complex lung disease characterized by the infiltration of cytokines secreted by various inflammatory cells. However, tolDCs have the potential to regulate immune cell response and promote the production of anti-inflammatory factors, thereby maintaining immune balance. While tolDC-based therapy has shown promise in treating RILI in recent studies, further research is required to determine its safety, optimal dosage, and treatment timing. At present, genetically modified organism (GMO) tolDCs have garnered significant interest from experts in the field and could potentially serve as the next advancement in the development of novel therapeutic strategies. In essence, tolDC therapy holds immense promise for the treatment of RILI.

## Author contributions

BL: Writing – original draft. YW: Writing – review & editing. GH: Writing – review & editing. MZ: Writing – review & editing.
